# The efficacy of stress reappraisal interventions on stress responsivity: A meta-analysis and systematic review of existing evidence

**DOI:** 10.1371/journal.pone.0212854

**Published:** 2019-02-27

**Authors:** Jenny J. W. Liu, Natalie Ein, Julia Gervasio, Kristin Vickers

**Affiliations:** 1 Department of Psychology, Ryerson University, Toronto, Ontario, Canada; 2 Institute for Stress and Wellbeing Research, Ryerson University, Toronto, Ontario, Canada; Peking University First Hospital, CHINA

## Abstract

**Background:**

The beliefs we hold about stress play an important role in coping with stressors. Various theoretical frameworks of stress point to the efficacy of reframing stress-related information through brief reappraisal interventions in order to promote adaptive coping.

**Purpose:**

The goal of the current meta-analysis and systematic review is to substantiate the efficacy of reappraisal interventions on stress responsivity compared to control conditions. Differences in experimental methodologies (e.g., type of stressor used, timing of reappraisal intervention, and content of intervention instructions) will be examined to further delineate their effects on intervention outcomes.

**Methods:**

The literature searches were conducted on May 16, 2018 using PsycINFO, ProQuest Dissertations and Theses, and PILOTS databases with no date restriction. The search terms included stress, reframing, reappraisal, mindset and reconceptualising. A total of 14 articles with 36 independent samples were included in the meta-analysis, while 22 articles with 46 independent samples were included in the systematic review. Random-effects model was used to test the null hypothesis using two-tailed significance testing. Fisher’s Z value was reported for each corresponding test. Heterogeneity tests are reported via Cochran’s Q-statistics.

**Results:**

Findings from both the meta-analysis and systematic review revealed that overall, reappraisal interventions are effective in attenuating subjective responsivity to stress. Standard differences in means across groups are 0.429 (*SE* = 0.185, 95% *CI* = 0.067 to 0.791; *z* = 2.320, *p* = .020). However, reappraisal intervention groups did not outperform control groups on measures of physiological stress, with standard differences of -0.084 (*SE* = 0.135, 95% *CI* = -0.349 to 0.180; *z* = -0.627, *p* = .531). Moderator analysis revealed heterogeneous effects suggesting large variability in findings.

**Conclusions:**

On one hand, findings may suggest a promising avenue for the effective management of self-reported stress and optimization of stress responses. However, more research is needed to better elucidate the effects, if any, of reappraisal interventions on stress physiology. Implications for the use of reappraisal interventions on stress optimization are discussed in the context of theoretical frameworks and considerations for future studies.

## Introduction

The pervasive societal attitude towards stress is the view that it is largely negative, with emphasis placed on the avoidance of stressors [1–3]. This view is largely rooted in the literature outlining the detrimental effects of stress on the human mind and body [4]. Stress has been illustrated to have both short-term and long-term effects, ranging from poor performance on stressor tasks in laboratory settings, to chronic illnesses and mortality rates in large-scale epidemiological studies [4–6].

Stress can be defined as a set of responses mounted in the presence of a perceived demand, threat, or challenge [7, 8]. These acute, short-term responses can serve an adaptive function, mobilizing energy and bodily resources and distributing them throughout the body where needed [9]. Continual activation of these responses, however, can deplete our bodies’ resources, resulting in wear and tear over time [10]. Emphasis placed solely on the elimination of stress may decrease the individual’s efforts and ability to become more resilient. While a large body of literature has amply documented the effects of stress on health and well-being [10], an emerging body of literature has begun to examine how shifting the beliefs we hold about acute experiences of stress may influence subsequent responses to stressors. Indeed, efforts have been made to introduce brief novel stress interventions in experimental settings with the goal of eliciting more adaptive acute responses to stressors [11–13].

### Theoretical framework of stress reappraisal

Many frameworks of stress and responsivity converge to suggest the efficacy for modifying, re-evaluating, or reframing beliefs about elements of stress through the use of reappraisal interventions. The Transactional Model of Stress and Coping emphasizes the process of appraisal and reappraisal in the evaluation of a stimulus as the primary step in mounting a potential stress response [7, 14]. The process of appraisal is also emphasized in cognitive emotion regulation as a way to effectively control affective, cognitive, and physiological outcomes [15, 16]. Indeed, individuals can be instructed to re-appraise a stimulus or event to experience more adaptive outcomes [16].

Shifts in thinking or beliefs to elicit more adaptive stress responses are also consistent with the theory of stress mindset [4]. Mindset refers to a set of beliefs an individual holds that act as lenses to guide future actions [17]. Researchers have experimented with alterations of stress mindsets through presentation of information about stress that emphasizes particular elements of the stress experience and outcomes [18].

Finally, shifting beliefs to elicit more adaptive stress response can also be distinguished by the Biopsychosocial Model of Challenge and Threat (BPS), whereby physiological profiles of stress responses effectively illustrate differences between adaptive and maladaptive coping [19]. Within BPS, clear distinctions are made between adaptive appraisal in response to a challenge (evaluation of high resources and low demands), and maladaptive appraisal in response to a threat (evaluation of low resources and high demand). These distinctions lie in the balance of acute responsivity with or without exertion of physiological regulatory efforts. Within this framework, individuals can be taught to reappraise their arousal to perceive the task as a challenge, rather than a threat [12].

### Stress reappraisal intervention across studies

A number of studies have examined the effects of brief reappraisal interventions on stress-related outcomes [6, 20–23]. Although diverse in their theoretical approach, methodology, and outcomes measured, each study has emphasized the potential impact of reappraising stress on eliciting more adaptive responses to stress.

For example, Jamieson et al. [21, 22] used a set of written instructions that asked participants to re-interpret their physiological stress response as signs of positive and successful coping. Outcome measures included both subjective and physiological measures, such as test performance, psychometric scales, and cardiovascular and endocrine outputs [21, 22]. These series of studies demonstrated better performance outcomes and attenuated stress responses in participants who received the reappraisal instructions.

In another approach, Crum et al. [4, 24] used three videos comprised of words, images, and music that were three minutes in length. To engender positive coping in response to stress, in these videos, stress was referred to as enhancing, such that pressure fuels performance. Outcome measures consisted of a series of questionnaires and neuroendocrine changes [4, 24]. The intervention successfully attenuated increases in salivary cortisol in participants undergoing stressful tasks.

Finally, Liu et al. [6] presented a balanced reframing of stress through a video, whereby both positive and negative effects of stress were presented to help individuals better cope with subsequent stressors. This study found that participants who watched the balanced reframing of stress video recovered more quickly to baseline values post-stressor, in comparison to those who did not receive the balanced information video on stress.

The large variabilities in design and methodology across studies complicate evaluation of the efficacy of reappraisal interventions. Despite existing evidence in support of their utility, several questions remain unanswered. First, given the heterogeneity across subjective and physiological measures of responsivity to stress [25], it is unclear whether reappraisal interventions are equally effective across different measures of stress. Another question is the timing of the intervention, and whether giving the reappraisal instructions before a stressor, or after a stressor, is maximally effective. A third question concerns whether different types of stressors are equally affected by the reappraisal interventions [26], specifically whether the effect of the reappraisal intervention is comparable in both active-type stressors (i.e., stressors requiring engagement and participation, such as performing a speech) and passive-type stressors (i.e., stressors that require minimal engagement on the part of participant, such as submerging hand in cold water). Finally, in approaching reappraisal interventions, the content of the reappraisal instructions can be domain-specific or domain-general [13]. Domain-specific refers to the tailoring of the intervention content to the expected responses that may be experienced during the upcoming stressor task (e.g., instructing participants to think about the sweaty palms and racing heart they will experience as indicators their bodies are readying for action). Domain-general interventions focus on shifting global views held on stress, without tailoring the language or content specific to any anticipated effects of upcoming stressors (e.g., informing participants of the beneficial effects of stress on individual wellbeing). To date, it remains unclear whether domain-specificity is a necessary criterion for the success of the brief reappraisal interventions. Taken together, the diversity in this research line (differences in stress outcomes, intervention timing, stressor type, and intervention instructions) thus merits further investigation about the extent to which reappraisal interventions improve stress coping.

### Rationale and aims

The goal of the current meta-analysis and systematic review is to substantiate the efficacy of brief reappraisal interventions on stress responsivity, compared to control conditions. Differences in experimental methodologies will be examined to further delineate their effects on experimental outcomes.

## Methods

### Eligibility criteria

The inclusion criteria for the current paper included: (1) studies that were written in English; (2) studies that included a reappraisal intervention condition; (3) studies that examined stress response measurements pre- and post-stressor (subjective and/or physiological); and (4) studies that used a validated stress-induction method that had been previously peer-reviewed and published (e.g., Trier Social Stress Test).

The exclusion criteria included the following: (1) review articles (including meta-analysis and systematic-reviews); (2) studies with animal populations; (3) studies that examined genome/genetic/allele studies; (4) studies with no stressors; (5) studies that included only individuals with medical and/or clinical conditions (for studies with both medical/clinical groups and a healthy control group; the healthy control groups were extracted for analysis); and (6) studies with children/youth (under age of 17) and older adult (over 60-year-old) populations.

### Search strategy

The meta-analysis and systematic review followed the procedures outlined on the Preferred Reporting Items for Systematic Review and Meta-Analysis (PRISMA) [27]. The literature searches were conducted on May 16, 2018 using PsycINFO, ProQuest Dissertations and Theses, and PILOTS databases with no date restriction. The search terms included *stress*, *reframing*, *reappraisal*, *mindset* and *reconceptualising*. Articles that included these terms were retained for review.

**Fig 1:**
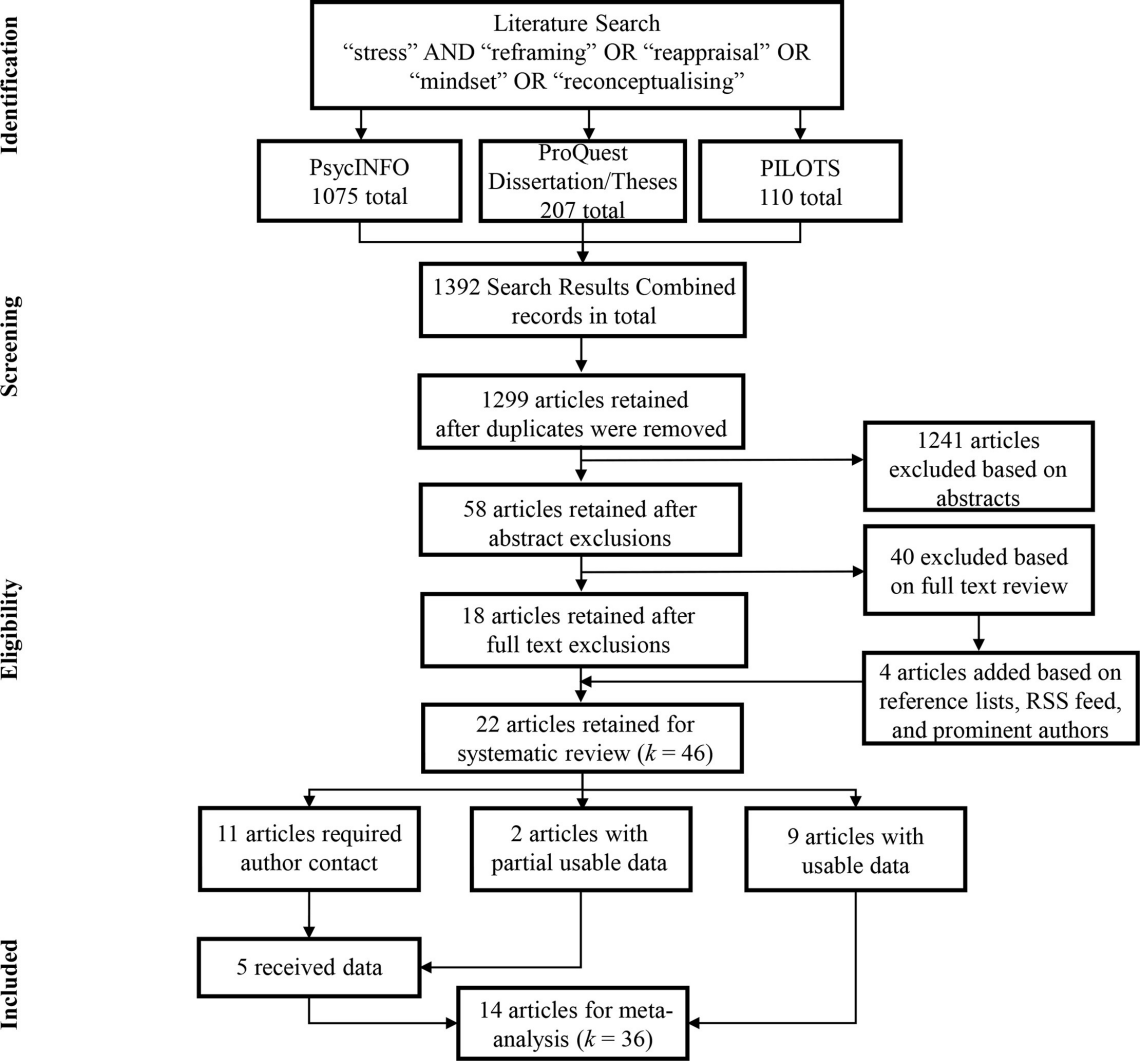
Preferred reporting items for systematic reviews and meta-analysis (PRISMA) flowchart.

The literature search resulted in a total of 1392 articles. After duplicates were removed, 1299 articles were retained. Upon reviewing titles and abstracts, 1241 articles were excluded based on the aforementioned criteria. Next, 58 articles received full text review. When reviewing the full texts, 40 articles were excluded with a total of 18 included for analysis. Additionally, 4 articles were added based on Rich Site Summary (RSS) Feed, Google Scholar notifications, and prominent authors’ personal websites. A total of 22 articles were included for analysis. Of these 22 articles, 9 provided extractable data (either in text or graphs), while 2 articles provided partial useable data. We extracted means and standard deviations for each time point (pre- and post-stressor). Additionally, we used Web Plot Digitizer (Rohatgi, https://automeris.io/WebPlotDigitizer/) to extract the means and standard errors for graphs and charts. The remaining 11 (and 2 partial) articles required author contact to obtain this information. Of these articles, 5 authors provided useable data. Overall, 14 articles were included for the meta-analysis, while 22 articles were included for the systematic review. Articles were reviewed by (J.L., N.E., and J.G.) and initial inter-rater reliability was 96%. Final article selection interrater reliability was 100%. All discrepancies were discussed with no disagreements between reviewers (Fig 1).

### Data extraction and coding

Demographic information, stress outcomes, and additional moderator information were extracted from each study. The demographic information included average age of the participants and percentage of female participants.

Stress measures included both subjective and physiological outcomes. Subjective outcomes included self-reported anxiety, stress, and affect (positive and negative). Physiological measures included salivary cortisol, salivary alpha-amylase, and cardiovascular outputs (heart rate, systolic blood pressure and diastolic blood pressure). For the meta-analysis, the following information was extracted between the intervention and control conditions: sample size and pre- and post-stressor means and standard deviations. We also extracted the types of measures used for subjective reports (e.g., visual analogue scales) and physiological (e.g., heart rate) responsivity. We then converted and standardized all values of salivary cortisol into unit measurements of nmol/L. For the systematic review, we also extracted the statistical findings on subjective and physiological responses for each article (e.g., a significant attenuation of the stress symptoms in the form of reduced responsivity pre- to post-stressor between intervention and control conditions).

Moderator information was also extracted from each article. We used four moderators for analysis. Moderator information included: type of stress outcome (subjective or physiological outcome), timing of intervention (reappraisal given before, or after the stressor), type of stressor (requiring active engagement or passive presence), and intervention instructions (content of reappraisal was domain-specific or domain-general).

### Data analytic plan for meta-analysis

Meta-analytic results were generated using the Comprehensive Meta-Analysis software (CMA) version 3.0 [28]. The effect sizes used were standard mean differences between intervention and control conditions [28]. The following information was extracted from each study and inputted into CMA: sample size, pre- and post-stressor means and standard deviations separated by condition (reappraisal and control) for each subjective outcome (subjective anxiety, stress, and affect [positive and negative]), and physiological outcome variables (salivary cortisol, heart rate, and blood pressure [systolic and diastolic]). Additionally, pre- and post-stressor correlations for each measure were required [28]. We estimated the pre‐ to post‐stressor Pearson’s correlation for four outcomes based on three studies [6, 29–30]: systolic blood pressure (*r* = 0.65), diastolic blood pressure, (*r* = 0.66), heart rate (*r* = 0.72), and subjective stress (*r* = 0.67). For subjective anxiety, the correlation was determined with one study (*r* = 0.96). For the remaining three outcomes (salivary cortisol and affect [positive and negative]), we did not receive any responses. Thus, a conservative correlation of *r* = 0.50 was used as the correlation coefficient for salivary cortisol and affect (positive and negative) due to lack of information [31–32]. It was also important to determine effect direction in relation to the hypothesis. Attenuation of responsivity in the reappraisal condition compared to control was regarded as having a positive effect direction that was consistent with study hypotheses. Differences across intervention and control conditions were standardized by change scores, with outcomes evaluated in standard differences in mean effects. Moderator information was also entered into CMA for analysis. A meta-regression was first conducted to determine the overall degree of influence of moderators on intervention effects, if any. Following this, moderators were used as sub-groups (as stated above) for subsequent independent analyses. Random-effects model was used to test the null hypothesis using two-tailed significance testing. Fisher’s Z value was reported for each corresponding test. Heterogeneity tests are reported via Cochran’s Q-statistics, which represents the total variance, defined as the sum of the squared deviations of each study [33]. Finally, publication bias was examined using visual funnel plots and Duval and Tweedie’s trim-and-fill procedure [34].

### Data analytic plan for systematic review

The reliability and validity of the methodologies used within each study were examine through study rigour ratings [35]. Each article was given a study rigour rating of weak, moderate, or strong. To assess study rigour, the following information was extracted and classified into five assessment categories: (1) sample size, (2) randomization of conditions, (3) use of subjective and physiological measures of stress response, (4) information about the intervention (description and theory), and (5) statistical testing [31].

For each study, numerical values were assigned to each of the assessment categorization categories stated above. A score of zero was assigned in each category if: (1) sample size was less than 10 participants per condition, (2) no control condition was used, (3) only subjective stress measures were used (given their ease of administration compared to the requirement for more complex methodological designs when incorporating physiological measures), (4) there was missing information about the intervention employed and theory behind the intervention, and (5) there was missing information about the statistical tests performed. A score of one was assigned in each category if: (1) the sample size was between 11 to 19 participants per condition, (2) partial/no randomization of the intervention and control conditions occurred, (3) only physiological stress measures were used, (4) there was some information about the intervention but missing theory, and (5) there was some incomplete information about the statistical tests performed. Lastly, a score of two was assigned in each category if: (1) the sample size was 20 or above per condition, (2) randomization of the intervention and control conditions occurred, (3) both subjective and physiological stress measures were used, (4) there was clear and detailed information about the intervention used and theory behind the intervention, and (5) there was clear/appropriate information about the statistical tests performed. After each of the assessment categories were recorded, the scores were tallied [31]. Studies received a rigour rating based on this tallied score: weak (total score between 0 to 5), moderate (total score between 6 to 7), or strong (total score of 8 or more; S1 Table).

## Results

### Study characteristics

Together, the systematic review and meta-analysis consisted of a total of 22 studies with 46 independent samples. These included a total of 2254 participants, with an overall percentage of 57% female, and with a mean age between 20–30 years across all studies. Within these studies, a total of 1118 participants were in the intervention condition, compared to 1136 participants in the control condition. The number of participants in each study’s groups ranged from 15 to 51 participants per study condition. A summary of study characteristics of each article is illustrated in Table 1 below.

**Table 1:** Summary of study characteristics.

Study	*N* (I/C)	Age	Sex (%F)	Type of Stressor	Timing of Intervention	Intervention Instructions	Subjective Outcome		Physiological Outcome	
							Pre-Stressor *M* (*SD*)	Post-Stressor *M* (*SD*)	Pre-Stressor *M* (*SD*)	Post-Stressor *M* (*SD*)
*Akinola et al. [36] Strong	51/46	24	59%	Active	Before	Domain-Specific	—	—	*I—*CORT 3.86 (1.93)	*I—*CORT 4.41 (3.03)
							—	—	*C–*CORT 3.86 (2.21)	*C–*CORT 4.41 (2.76)
Beltzer et al. [37] Strong	42/43	25	66%	Active	Before	Domain-Specific	—	—	AMY (*I* > *C*)– significant increase	
							—	—		
*Bowlin [38] Strong	26/27	-	51%	Passive	Before	Domain-General	*—*	*—*	*I—*SBP 109.50 (9.34)	*I—*SBP 109.03 (11.1)
							*—*	*—*	*C–*SBP 112.05 (12.00)	*C–*SBP 109.88 (11.22)
							—	—	*I—*DBP 64.53 (5.27)	*I—*DBP 63.70 (5.59)
							—	—	*C–*DBP 64.80 (6.48)	*C–*DBP 64.71 (6.93)
							—	—	*I—*HR 66.34 (10.55)	*I—*HR 69.21 (8.93)
							—	—	*C–*HR 68.95 (13.77)	*C–*HR 71.59 (13.98)
Brooks [39] Moderate	-	20	57%	Active	Before	Domain-Specific	ANX (*I* vs *C*)– non-significant		HR (*I* vs *C*)– non-significant	
Cohen & Mor [40] Moderate	42/45	24	63%	Passive	After	Domain-Specific	VAS-NEG (*I* > *C*)– significant decrease		—	—
									—	—
*Crum et al. [24] Strong	30/30	24	65%	Active	Before	Domain-General	*I–*PANAS-P 3.10 (0.66)	*I–*PANAS-P 2.80 (0.77)	*I–*CORT 0.05 (0.02)	*I—C*ORT 0.07 (0.04)
							*C–*PANAS-P 3.04 (0.80)	*C–*PANAS-P 2.58 (0.91)	*C–*CORT 0.03 (0.02)	*C–*CORT 0.07 (0.07)
							*I–*PANAS-N 1.52 (0.47)	*I–*PANAS-N 2.06 (0.67)	—	—
							*C–*PANAS-N 1.58 (0.56)	*C–*PANAS-N 2.14 (0.76)	—	—
*Denson et al. [41] Strong	45/45	21	52%	Active	Before	Domain-Specific	*I–*PANAS-P 2.68 (0.89)	*I–*PANAS-P 2.32 (0.97)	*I–*CORT 9.05 (26.71)	*I–*CORT 13.35 (46.57)
							*C–*PANAS-P 2.57 (0.75)	*C–*PANAS-P 2.19 (0.76)	*C–*CORT 8.09 (25.77)	*C–*CORT 10.22 (46.57)
							*I–*PANAS-N 1.33 (0.37)	*I–*PANAS-N 1.69 (0.60)	*I–*HR 75.29 (15.70)	*I–*HR 95.58 (16.91)
							*C–*PANAS-N 1.61 (0.51)	*C–*PANAS-N 1.91 (0.81)	*C–*HR 77.09 (14.43)	*C–*HR 99.69 (17.78)
	42/45	21	57%	Passive	Before	Domain-Specific	—	—	*I–*CORT 8.15 (4.51)	*I–*CORT 8.82 (5.71)
							—	—	*C–*CORT 7.56 (4.03)	*C–*CORT 6.91 (5.50)
							—	—	*I–*HR 75.74 (8.81)	*I–*HR 73.68 (10.89)
							—	—	*C–*HR 77.66 (10.94)	*C–*HR 72.35 (7.72)
*Erazo [42] Strong	28/34	22	77%	Active	Before	Domain-General	*I–*VAS-STR 380.32 (70.91)	*I–*VAS-STR 177.73 (95.27)	CORT (*I* > *C*)– significant decrease	
							*C–*VAS-STR 379.07 (63.36)	*C–*VAS-STR 454.24 (58.60)		
							*I–*VAS-ANX 11.35 (4.43)	*I–*VAS-ANX 17.64 (4.56)	—	—
							*C–*VAS-ANX 12.04 (5.59)	*C–*VAS-ANX 19.85 (3.82)	—	—
*Germain & Kangas [43] Moderate	34/34	30	-	Passive	After	Domain-Specific	*—*	*—*	*I–*SBP 117.56 (13.77)	*I–*SBP 119.68 (15.09)
							—	—	*C–*SBP 121.14 (18.73)	*C–*SBP 129.50 (19.31)
							—	—	*I–*DBP 72.94 (11.22)	*I–*DBP 77.65 (11.99)
									*C–*DBP 74.72 (12.84)	*C–*DBP 80.53 (13.86)
*Gong et al. [44] Moderate	21/21	20	83%	Active	Before	Domain-Specific	I–SAI 33.10 (6.00)	I–SAI 37.10 (6.75)	—	—
							C–SAI 33.24 (4.44)	C–SAI 39.00 (6.20)	—	—
Gross [16] Strong	40/40	21	50%	Passive	Before	Domain-Specific	AFF (*I* > *C*)– significant increase		HR (*I* > *C*)– significant decrease	
Jamieson et al. [21] Moderate	-	-	48%	Active	Before	Domain-Specific	—	—	AMY (*I* > *C*)– significant increase	
							—	—		
*Jamieson et al. [12] Strong	15/15	22	5%	Active	Before	Domain-Specific	*I–*PANAS-P 2.39 (.71)	*I–*PANAS-P 2.48 (.79)	HR-VR (*I* > *C*)– significant decrease	
							*C–*PANAS-P 2.52 (.68)	*C–*PANAS-P 2.22 (.63)		
							*I–*PANAS-N 1.84 (.88)	*I–*PANAS-N 1.69 (.79)	—	—
							*C–*PANAS-N 1.90 (.78)	*C–*PANAS-N 2.03 (.74)	—	—
Jamieson et al. [22] Moderate	18/18	26	62%	Active	Before	Domain-Specific	—	—	HR-VR (*I* > *C*)– significant decrease	
							—	—		
Kneeland et al. [45] Weak	-	22	67%	Active	Before	Domain-General	PANAS-P (*I* > *C*)– significant decrease		—	—
									—	—
							PANAS-N (*I* vs *C*)– non-significant		—	—
									—	—
							STAI (*I* vs *C*)– non-significant		—	—
									—	—
*Liu et al. [6] Strong	15/15	21	70%	Active	Before	Domain-General	*I–*VAS-STR 44.13 (15.93)	*I–*VAS-STR 47.00 (18.46)	*I–*SBP 129.94 (29.78)	*I–*SBP 124.86 (29.71)
							*C–*VAS-STR 52.00 (28.89)	*C–*VAS-STR 60.20 (25.39)	*C–*SBP 129.80 (15.88)	*C–*SBP 128.41 (25.31)
							—	—	*I–*DBP 76.98 (15.68)	*I–*DBP 65.88 (16.66)
							—	—	*C–*DBP 78.37 (17.26)	*C–*DBP 74.81 (17.83)
							—	—	*I–*HR 96.50 (20.07)	*I–*HR 87.60 (20.42)
							—	—	*C–*HR 96.52 (16.23)	*C–*HR 95.23 (19.36)
	15/15	21	70%	Passive	Before	Domain-General	*I–*VAS-STR 37.73 (26.76)	*I–*VAS-STR 36.13 (23.69)	*I–*SBP 115.91 (13.87)	*I–*SBP 115.62 (13.66)
							*C–*VAS-STR 37.20 (25.91)	*C–*VAS-STR 35.73 (26.80)	*C–*SBP 127.50 (18.96)	*C–*SBP 126.52 (19.79)
							—	—	*I–*DBP 68.29 (7.37)	*I–*DBP 65.19 (9.47)
							—	—	*C–*DBP 75.96 (11.10)	*C–*DBP 72.41 (12.21)
							—	—	*I–*HR 87.19 (10.00)	*I–*HR 83.14 (10.58)
							—	—	*C–*HR 91.73 (11.48)	*C–*HR 88.97 (13.07)
Moore et al. [46] Moderate	-	20	44%	Active	Before	Domain-Specific	—	—	HR (*I* vs *C*)– non-significant	
							—	—		
*Popham [47] Strong	33/33	28	49%	Active	Before	Domain-Specific	*I–*VAS-STR 34.20 (16.55)	*I–*VAS-STR 38.14 (33.40)	*—*	*—*
							*C–*VAS-STR 36.98 (17.66)	*C–*VAS-STR 33.81(21.24)	*—*	*—*
							*I–*VAS-POS 33.85 (16.24)	*I–*VAS-POS 25.49 (22.62)	*—*	*—*
							*C–*VAS-POS 26.65 (17.85)	*C–*VAS-POS 19.28 (23.82)	*—*	*—*
*Sammy et al. [48] Strong	28/26	22	39%	Active	Before	Domain-Specific	*I–*IAMS 3.12 (1.69)	*I–*IAMS 2.76 (1.48)	*I–*HR 70.23 (9.46)	*I–*HR 71.25 (9.13)
							*C–*IAMS 2.53 (1.18)	*C–*IAMS 2.59 (1.21)	*C–*HR 74.15 (13.09)	*C–*HR 70.82 (12.48)
*Wang et al. [49] Weak	28/27	23	49%	Active	Before	Domain-Specific	*I–*STAI 1.11 (1.34)	*I–*STAI 2.29 (1.82)	—	—
							*C–*STAI 1.48 (1.45)	*C–*STAI 3.07 (2.35)	—	—
*Woud et al. [50] Moderate	37/37	22	51%	Passive	After	Domain-General	*I–*VAS-NEG 1.73 (1.22)	*I–*VAS-NEG 3.74 (1.90)	—	—
							*C–*VAS-NEG 1.64 (1.08)	*C–*VAS-NEG 3.99 (2.08)	—	—
*Zhan et al. [51] Moderate	30/30	21	66%	Passive	Before	Domain-General	*I–*STR 3.63 (0.11)	*I–*STR 2.63 (0.43)	*I–*CORT 13.79 (2.10)	*I–*CORT 27.2 (5.30)
							*C–*STR 3.63 (0.14)	*C–*STR 2.87 (0.53)	*C–*CORT 13.68 (1.63)	*C–*CORT 16.72 (1.79)

*Notes*. I = intervention group; C = control condition; CORT = salivary cortisol (nmol/L); AMY = salivary alpha-amylase (units/mL); HR = heart rate (bpm); HR—VR = heart rate—vascular resistance SBP = systolic blood pressure (mm/Hg); DBP = diastolic blood pressure (mm/Hg); ANX = anxiety without defined scale; STR = stress without defined scale; AFF = affect (positive and negative combined); PANAS-N = positive and negative affect schedule—negative; PANAS-P = positive and negative affect scale—positive; VAS-NEG = visual analog scale-negative affect; VAS-POS = visual analog scale—positive; VAS-STR = visual analog scale—stress; VAS-ANX = visual analog scale—anxiety; SAI = Chinese version of Revised State Anxiety, from the State Trait Anxiety Inventory; STAI = The State-Trait Anxiety Inventory; IAMS = immediate anxiety measurement scale

* = studies that are included in the meta-analysis; decreased = pre- to post-stressor changes were less in the intervention condition compared to the control condition; increased = pre- to post-stressor changes were more in the intervention condition compared to the control condition.

### Meta-analysis

A total of 14 studies with 36 independent samples were included in the current meta-analysis. Of these samples, 17 measured subjective outcomes, including 4 that measured anxiety, 5 measuring stress, and 8 measuring affect. Nineteen samples measured physiological outcomes, including 5 samples measuring salivary cortisol, 6 measuring heart rate, 4 samples measuring systolic blood pressure, and 4 measuring diastolic blood pressure.

### Reappraisal intervention versus control conditions

To examine the effects of reappraisal on stress outcome measures (subjective stress, subjective anxiety, subjective affect, salivary cortisol, heart rate, and blood pressure), an overall comparison was conducted between the pre- and post-stressor outcomes across reappraisal and control conditions. A total of 36 samples were entered into a random effects model. Results indicate that the reappraisal conditions and the control conditions did not differ significantly pre- to post-stressor, with a standard difference in means of 0.15 (*SE* = 0.114, 95% *CI* = -0.073 to 0.372; *z* = 1.319, *p* = .187). Further, these effects were heterogeneous, *Q*(35) = 213.15, *p* < .001, suggesting that samples had large variability of scores.

In addition to examining the efficacy of reappraisal intervention on stress responsivity outcomes, the effects of possible moderators were also examined. A backwards step meta-regression with moment method and random effects model was used. Potential moderators (type of outcomes, timing of intervention, type of stressor, and intervention instructions) were entered into a linear regression model. *Q*-statistics determined that these potential moderators significantly related to the effect size differences across reappraisal and control conditions, *Q*(4) = 16.23, *p* = .003. Study moderators together accounted for 11% of the variance in differences in responsivity to stressor between reappraisal and control conditions. Results indicated that types of study outcomes assessed (subjective versus physiological, *p* = .04) and type of stressor (active versus passive, *p* < .001) accounted for significant differences in experimental effects across conditions. The timing of interventions (*p* = .13), and intervention instructions (*p* = .15) did not account for significant differences across studies. As such, subsequent analyses focused on types of outcomes and types of stressors only.

### Types of study outcomes as moderator

Response to stressors were measured in two domains, subjective and physiological outcomes. Based on the results of the meta-regression, type of study outcome significantly affected group differences between intervention and control conditions. Follow-up meta-analyses were conducted to explore differences in study outcomes.

#### Subjective outcomes to stressors

To examine the effects of reappraisal on subjective measures (stress, anxiety, and affect) compared to control, an overall comparison was conducted between the pre- and post-stressor outcomes across study samples. A total of 17 samples were entered into a random effects model. Results indicated that the reappraisal conditions outperformed the control conditions pre- to post-stressor, with a standard difference in means of 0.429 (*SE* = 0.185, 95% *CI* = 0.067 to 0.791; *z* = 2.320, *p* = .020). These effects were heterogeneous, *Q*(16) = 118.374, *p* < .001, suggesting that samples had a large variability of scores. See Table 2 for subjective measures separated by outcomes.

**Table 2:** Subjective measures separated by outcome.

Outcome Variable	*k*	Standard difference in means	*SE*	95% *CI*		*z*	*p*	*Q*
				Lower	Upper			
**Anxiety**	4	0.770	0.143	0.491	1.050	5.403	**< .001**	1.803
*Active*	4	0.770	1.43	0.497	1.050	5.403	**< .001**	1.803
**Stress**	5	0.753	0.716	-0.650	2.156	1.052	.293	95.977*
*Active*	3	1.492	1.323	-1.101	4.085	1.127	.260	82.864*
**Affect**	8	0.090	0.090	-0.085	0.266	1.08	.314	3.675
*Active*	7	0.072	0.097	-0.118	0.262	0.741	.458	3.434

Note.

*p < .05

**Fig 2:**
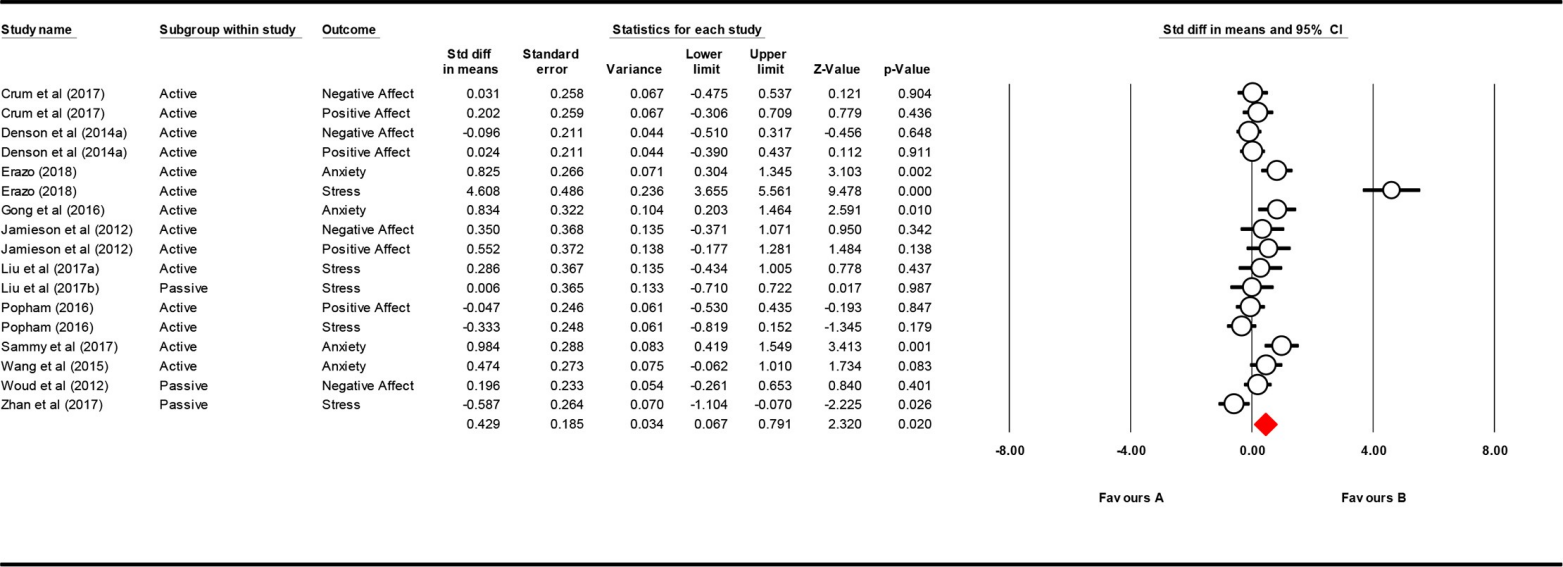
Forest plot for subjective outcomes.

Visual inspection of the forest plot (see Fig 2) suggested that the stress scores from Erazo may have especially impacted the effects of the overall meta-analysis [42]. Thus, a secondary analysis examined the overall effects of reappraisal compared to control on measures of stress, anxiety and affect. A total of 16 samples were entered into a random effects model excluding Erazo [42], with results indicating that the reappraisal conditions continued to outperform the control conditions pre- to post-stressor, but these results were trending. Findings indicated a standard difference in means of 0.206 (*SE* = 0.108, 95% *CI* = -0.004 to 0.417; *z* = 1.918, *p* = .055). These effects were heterogeneous, *Q*(15) = 36.656, *p* = .001, suggesting that samples also had a large variability of scores.

#### Physiological outcomes to stressors

To examine the effects of reappraisal on physiological outcomes (heart rate, blood pressure, and cortisol), an overall comparison between the pre- and post-stressor outcomes across study samples was conducted. A total of 19 samples were entered into a random effects model. Results indicated that the reappraisal conditions did not outperform the control conditions pre- to post-stressor. Effects indicated a standard difference in means of -0.084 (*SE* = 0.135, 95% *CI* = -0.349 to 0.180; *z* = -0.627, *p* = .531). These effects were heterogeneous, *Q*(18) = 82.860, *p* < .001, suggesting that samples had a large variability of scores.

### Types of stressors as moderator

Results from the meta-regression also identified that the type of stressors (active versus passive) significantly affected group differences between intervention and control. To further explore these differences, follow-up meta-analyses were conducted between active and passive stressors, as well as in subjective and physiological stress outcomes.

#### Passive stressors

First, the effects of all stress outcomes (subjective and physiological) were examined for experiments that used a passive type of stressor. A total of 14 samples were entered into a random effects model. Results indicated that within studies that used a passive stressor, the reappraisal conditions did not outperform the control conditions pre- to post-stressor, with a standard difference in means of -0.227 (*SE* = 0.177, 95% *CI* = -0.573 to 0.120; *z* = -1.284, *p* = .199). These effects were heterogeneous, *Q*(13) = 73.094, *p* < .001, suggesting that samples had a large variability of scores.

A further examination considered the interactive effects of passive stressor with subjective outcomes (anxiety, stress and affect). Three samples were entered into a random effects model, with results indicating that within these samples, no group differences were detected between the intervention and control, with a standard difference in means of -0.130 (*SE* = 0.260, 95% *CI* = -0.639 to 0.379; *z* = -0.501, *p* = .616). Within these samples, effects were not heterogeneous, *Q*(2) = 5.088, *p* = .079. Due to low number of study sample, subjective outcomes were unable to be separated for subsequent analysis.

For studies that used a passive stressor, the physiological outcomes were not significantly different across intervention and control, with a standard difference in means of -0.258 (*SE* = 0.220, 95% *CI* = -0.688 to 0.173; *z* = -1.174, *p* = .241). Within these 11 samples, the effects were found to be heterogeneous, *Q*(10) = 67.878, *p* < .001.

#### Active stressors

We then examined the effects of all stress outcomes (subjective and physiological) for experiments that used an active stressor. A total of 22 samples were entered into a random effects model. Results indicated that within studies using an active stressor, the reappraisal conditions did outperform the control conditions pre- to post-stressor, with a standard difference in means of 0.388 (*SE* = 0.141, 95% *CI* = 0.112 to 0.336; *z* = 2.753, *p* = .006). These effects were heterogeneous, *Q*(21) = 120.780, *p* < .001, suggesting that samples had a large variability of scores.

For studies with active stressors, a meta-analysis of 14 subjective outcomes (stress, anxiety and affect) found that the intervention conditions outperformed the control conditions, with a standard difference in means of 0.556 (*SE* = 0.215, 95% *CI* = 0.135 to 0.978; *z* = 2.587, *p* = .010). These effects were heterogeneous, *Q*(13) = 106.424, *p* < .001, suggesting that samples had a large variability of scores. See Table 2 for subjective measures separated by outcome.

Finally, a meta-analysis on studies with active stressors was conducted that examined group differences in eight samples of physiological outcomes. Results from a random effects model found no significant differences between the intervention and control conditions, with a standard difference in means of 0.106 (*SE* = 0.113, 95% *CI* = -0.116 to 0.328; *z* = 0.937, *p* = .349). Within these samples, the effects were not found to be heterogeneous, *Q*(7) = 10.045, *p =* .186.

### Publication bias

Duval and Tweedie’s trim-and-fill procedure was used to estimate the possible presence of publication bias in these findings [34]. A visual inspection estimation of the funnel plot suggested that the sampled studies were evenly clustered towards the peak, and nested within both sides of the funnel plot. Point estimates of overall effect sizes for observed and adjusted values were identical to the right of the mean, and relatively similar to the left, with an observed value of 0.43 CI (0.22, 0.64), and adjusted value at 0.20 CI (-0.01, 0.42). Fail-safe analysis revealed that it would take 707 studies with nonsignificant findings to nullify the results of the current meta-analyses, such that the combined 2-tailed p-value would exceed 0.05. In other words, there would need to be 29.5 missing studies for every observed study for the effect to be nullified.

### Systematic review

A total of 22 articles with 46 independent samples were included in the systematic review. Six samples measured salivary cortisol, 2 samples measured alpha amylase, 11 samples measured heart rate, 4 samples measured systolic blood pressure and diastolic blood pressure, 5 samples measured subjective stress, 6 samples measured subjective anxiety, and 8 samples measured subjective affect. Statistical findings that indicated whether results attenuated responsivity (confirming reappraisal was effective), did not attenuate responsivity (no difference across groups), or were nonsignificant (no statistically significant change) were used for each outcome measure. Of note, these comparisons were direct comparisons of mean differences, which were Bonferroni-corrected, and thus much more conservative than multiple comparisons reported in the original published articles [52, 53]. Study rigour was used to analysis and compare the systematic review with the meta-analysis results. Table 3 summarizes the findings results of the systematic review.

**Table 3:** Systematic review results summary for each outcome variables from pre- to post-stressor.

Outcome Variable	Author	Rigour	*N* (I/C)	Stressor Type	Intervention Instruction	Results
**Subjective Stress**						
	*Erazo [42]	Strong	28/34	Active	General	I < C–Attenuated
	*Liu et al [6a]	Strong	15/15	Active	General	I vs C—Nonsignificant
	*Liu et al [6b]	Strong	15/15	Passive	General	I vs C—Nonsignificant
	*Popham [47]	Strong	33/33	Active	Specific	I vs C—Nonsignificant
	*Zhan et al [51]	Moderate	30/30	Passive	General	I > C–Not Attenuated
**Subjective Anxiety**						
	Brooks [39]	Moderate	-	Active	Specific	I vs C—Nonsignificant
	*Erazo [42]	Strong	28/34	Active	General	I < C–Attenuated
	*Gong et al [44]	Moderate	21/21	Active	Specific	I < C–Attenuated
	Kneeland et al [45]	Weak	-	Active	General	I vs C–Nonsignificant
	*Sammy et al [48]	Strong	28/26	Active	Specific	I < C–Attenuated
	*Wang et al [49]	Weak	28/27	Active	Specific	I vs C–Nonsignificant
**Subjective Affect**						
	Cohen & Mor [40]	Moderate	42/45	Passive	Specific	I > C–Not Attenuated^b^
	*Crum et al [24]	Strong	30/30	Active	General	I vs C–Nonsignificant
	*Denson et al [41a]	Strong	45/45	Active	Specific	I vs C–Nonsignificant
	Gross [16]	Strong	40/40	Passive	Specific	I > C–Attenuated^c^
	*Jamieson et al [12]	Strong	15/15	Active	Specific	I vs C–Nonsignificant
	Kneeland et al [45]	Weak	-	Active	General	I > C–Attenuated^d^
	*Popham [47]	Strong	33/33	Active	Specific	I vs C–Nonsignificant
	*Woud et al [50]	Moderate	37/37	Passive	General	I vs C–Nonsignificant
**Salivary Cortisol**						
	*Akinola et al [36]	Strong	51/46	Active	Specific	I vs C–Nonsignificant
	*Crum et al [24]	Strong	30/30	Active	General	I vs C–Nonsignificant
	*Denson et al [41a]	Strong	45/45	Active	Specific	I vs C–Nonsignificant
	*Denson et al [41b]	Strong	42/45	Passive	Specific	I vs C–Nonsignificant
	Erazo [42]	Strong	28/34	Active	General	I < C–Attenuated
	*Zhan et al [51]	Moderate	30/30	Passive	General	I < C–Attenuated
**Alpha Amylase**						
	Beltzer et al [37]	Strong	42/43	Active	Specific	I > C–Not Attenuated
	Jamieson et al [21]	Moderate	-	Active	Specific	I > C–Not Attenuated
**Heart Rate**						
	*Bowlin [38]	Strong	26/27	Passive	General	I vs C–Nonsignificant
	Brooks [39]	Moderate	-	Active	Specific	I vs C—Nonsignificant
	*Denson et al [41a]	Strong	45/45	Active	Specific	I vs C—Nonsignificant
	*Denson et al [41b]	Strong	42/45	Passive	Specific	I > C–Not Attenuated
	Gross [16]	Strong	40/40	Passive	Specific	I vs C—Nonsignificant
	Jamieson [12]^a^	Strong	15/15	Active	Specific	I < C—Attenuated
	Jamieson [22] ^a^	Moderate	18/18	Active	Specific	I < C—Attenuated
	*Liu et al [6a]	Strong	15/15	Active	General	I vs C—Nonsignificant
	*Liu et al [6b]	Strong	15/15	Passive	General	I vs C—Nonsignificant
	Moore et al [46]	Moderate	-	Active	Specific	I vs C—Nonsignificant
	*Sammy et al [48]	Strong	28/26	Active	Specific	I vs C—Nonsignificant
**Systolic Blood Pressure**						
	*Bowlin [38]	Strong	26/27	Active	General	I vs C—Nonsignificant
	*Germain & Kangas [43]	Moderate	34/34	Passive	Specific	I vs C—Nonsignificant
	*Liu et al [6a]	Strong	15/15	Active	General	I vs C—Nonsignificant
	*Liu et al [6b]	Strong	15/15	Passive	General	I vs C—Nonsignificant
**Diastolic Blood Pressure**						
	*Bowlin [38]	Strong	26/27	Passive	General	I vs C—Nonsignificant
	*Germain & Kangas [43]	Moderate	34/34	Passive	Specific	I vs C—Nonsignificant
	*Liu et al [6a]	Strong	15/15	Active	General	I vs C—Nonsignificant
	*Liu et al [6b]	Strong	15/15	Passive	General	I vs C—Nonsignificant

*Notes*. I = intervention condition; C = control condition; decreased = pre- to post-stressor changes were less in the intervention condition compared to the control condition; increased = pre- to post-stressor changes were more in the intervention condition compared to the control condition

^a^ = these studies used measures of cardiac output and total peripheral resistance, which are measures of regulatory mechanism

^b^ = this study only explained negative affect

^c^ = this study found that the intervention significantly increased positive affect and significantly decreased negative affect

^d^ = this study found that the intervention significantly decreased positive affect; negative affect was not significant

* = articles in the meta-analysis

### Subjective measure outcomes

For the subjective measures (stress, anxiety and affect), 8 out of 19 samples showed significant differences between the intervention and control conditions. Six of these samples found that the intervention condition resulted in an attenuation of subjective responsivity to stress, as hypothesized. The majority of these studies were given a strong rigour rating (4 out of 6). The remaining two samples found opposite experimental effects. However, these studies were rated moderate in rigour, suggesting less weight should be attributed to these findings. The remaining 11 samples measuring subjective outcomes reported non-significant findings. The majority (8 out of 11) of the nonsignificant samples were with subjective stress and affect measures. A majority of studies that found significant findings were specific to the domain of anxiety (3 out of 6), which is consistent with findings of the meta-analysis. These three studies were primarily strong in rigour (2 out of 3 strong, 1 moderate). As for the remaining three anxiety samples, they reported non-significant findings. However, these samples were predominately weak in rigour (2 out of 3). This again supports the findings of the meta-analysis. Consistent with the meta-analysis findings, the significant effects were exclusively for subjective anxiety, while the subjective stress and affect did not show significant effects. It is important to note that all significant findings pertaining to anxiety utilized an active stressor type, in which participants were required to actively engage in participation during the stressor task.

### Physiological measure outcomes

The majority of the samples (20 out of 27) that reported physiological data (salivary cortisol, heart rate, and blood pressure) were non-significant. All of these studies were rated moderate to strong in rigour, suggesting their findings should be given more weight. The other 7 samples reported significant findings. Two samples examined regulatory mechanisms (e.g., cardiac output) that were not otherwise explained in any other study nor examined through our meta-analysis. Of the other five samples, two confirmed the experimental hypotheses, and were rated moderate and strong in rigour. The other three samples that found the opposite findings to the hypothesis were given strong and moderate rigour scores. Given that the majority of the samples found non-significant differences in physiological data, these findings support the results of the meta-analysis. Due to the limited number of samples that measured salivary alpha-amylase, this outcome was able to be examined only in the context of the systematic review. Both samples that measured salivary alpha-amylase found that the intervention condition reported a higher increase in values post-stressor compared to the control condition. These studies were rated moderate and strong in rigour.

## Discussion

The current meta-analysis and systematic review examined the efficacy of brief, single-session stress reappraisal interventions on attenuating stress responses compared to control conditions. A test of omnibus effects of the reappraisal compared to the control did not find an overall difference across conditions when combining all measures of stress responsivity. However, further exploration of differences in experimental methodologies yielded results that merited further scrutiny, with regards to both the type of study outcomes (subjective versus physiological outcomes of stress) and the type of stressor (active versus passive). Altogether, experimental variations across studies examined through study moderators accounted for a small portion of the overall variances across results. Of these moderators, the type of outcomes assessed and the type of stressor used were found to be driving some of the experimental findings. Specifically, subjective outcomes in the reappraisal conditions outperformed the control conditions pre- to post-stressor. Additionally, the reappraisal conditions within studies using an active stressor outperformed the control conditions pre- to post-stressor in the subjective outcomes.

The systematic review of study rigour indicated that majority of the studies were moderate or robust in study rigour. In addition, outcomes reported in the systematic review were largely consistent with results of the meta-analysis, such that significant findings were only found within the subjective measures. Taken together, findings from both the meta-analysis and systematic review suggest that the reappraisal intervention can be effective in attenuating subjective outcomes of stress compared to a control condition. However, the reappraisal interventions did not differ from the control condition in reducing or attenuating physiological responsivity. Further, the efficacy of the reappraisal studies appears to be limited to studies employing an active-type stressor.

The findings across subjective outcomes within active stressor study paradigms are thus an important consideration for the efficacy of reappraisal interventions. These findings across both the meta-analysis and systematic review are supported and explained by the framework of the Biopsychosocial Model of Challenge and Threat (BPS) [19, 54]. Within this model, the distinctions between responses to stressors in a challenge context versus a threat context are clearly articulated through both subjective and physiological outcomes.

### The BPS model of challenge and threat in the context of reappraisal interventions

Within the BPS framework, the appraisal (and reappraisal) process(es) in response to a novel stimulus are emphasized. The resource versus demand evaluation is the key indicator between an activation of physiological response pathway that represents a challenge response, versus that of a threat response. Consistent with the Transactional Model of stress, the appraisal process requires an individual to evaluate whether the novel stimulus requires high versus low demands, and whether the individual perceives he or she has enough resources to cope with such demands [7, 19, 54].

The underlying assumption within both models is the engagement an individual will have with the perceived stimulus or stressor. The BPS model and its subsequent distinct trajectories of stress response are predicated on an individual appraising the stressor as a task that requires task engagement [19]. Given this premise, it may be clear why the efficacy of the reappraisal interventions was specific only to studies using an active-type stressor, in which engagement with the task was required in order to participate. Within passive-type stressor studies, such as instructing participants to watch a sad film, or submerge their hand in a bucket of water, participants may not have engaged at all with the task enough to appraise it as either a challenge, nor a threat. Without the appraisal of a task as requiring attentional demand and resource allocation, there is no further need for engagement. Within these instances, reappraisal interventions would not be useful as there would be no perceived need to engage with the demands of any task.

Task engagement results in effort, the marker of distinction between trajectories of challenge-based coping versus threat-based coping [19, 54]. Despite its emphasized importance, few studies employing the reappraisal interventions used any measures of effort in the evaluation of stress outcomes. Indeed, only two studies [21, 22] examined regulatory efforts through cardiovascular outputs and vascular resistance. Within these two studies, the systematic review found that both studies reported significant attenuation effects of the reappraisal intervention compared to that of a control condition. These findings importantly underscore the need to examine regulatory mechanisms that are consistent with the BPS model.

### Considerations for measurement selection in stress studies

The finding of the stress-attenuating effects of reappraisal interventions only in studies that used active stressors, and exclusively for measures of subjective anxiety, may be more indicative of the lack of appropriate stress outcome measures employed across studies. As stated above, of the 22 studies and 46 samples collected, only two studies effectively measured regulatory mechanisms of stress. Yet, central to the efficacy of the reappraisal interventions is the BPS model, which distinguishes between challenge and threat on task engagement and effort. Indeed, the authors of the BPS model have emphasized that simple cardiovascular measures of blood pressure and heart rate alone are insufficient in capturing effortful regulation [54]. The authors assert that few differences in cardiovascular output are expected across study conditions. Instead, the outputs are more indicative of the relative metabolic demands of various stressor tasks [54, 55]. The key mechanism of task demand and appraisal that is targeted by these reappraisal interventions is the need to regulate arousal through perceived task demand. Thus, studies employing the reappraisal interventions need to mindfully measure these regulatory mechanisms in order to detect meaningful differences across conditions.

In addition, the use and presentation of validated, psychometric scales versus visual analogue scales may have contributed to the distinctions in efficacy between subjective anxiety and subjective stress. All studies measuring subjective stress levels employed a visual analogue scale (VAS) type measure to capture changes in self-reported stress levels pre- and post-stressor. The use of VAS-type measures has been shown to be effective in capturing changes in unidimensional constructs, such as localized pain and general anxiety [56, 57]. However, these scales were administered multiple times within the span of a single laboratory visit, while previous studies examined meaningful changes over the span of days across various types of interventions and drug therapies. In addition, in a study that directly assessed the efficacy of VAS versus numerical rating scales, results highlighted that the numerical rating scales were more sensitive to detect acute changes [58].

Indeed, subjective differences across intervention and control conditions in response to the reappraisal intervention were limited to measures of anxiety, where a majority of the studies (4 out of 6 overall, and 2 out of 3 that found statistical significance) employed validated, multi-dimensional measures of anxiety using psychometric scales that consisted of more than a single question. Although there is debate on the representativeness of various types of anxiety scales in assessing dimensions of anxiety [59–61], the use of multiple items on multidimensional scales and subscales offer a more comprehensive assessment of changes that may not otherwise be captured when using a single VAS repeatedly during the course of a short experiment. Taken together, these findings suggest that stress outcome measurements should be grounded in theoretical and practical importance, and driven by the need to detect meaningful distinctions through mechanisms of change.

### Challenges and conclusion

These considerations noted, it should be emphasized that differences in study methodologies assessed accounted for only a conservative portion (11%) of the variability across study outcomes. This highlights the complexity of the individual stress responses and the challenges stress researchers may face when designing studies to capture group differences in responsivity to stress. Further, it is also possible that other factors not assessed across studies may have contributed to differences in the effects of reappraisal interventions compared to a control condition. Future studies should consider the importance of experimental variations, such as length of experiment, length and content of stressors used, content of intervention and understanding of reappraisal instructions, among other factors. Further, limitations of the diversity of stress outcome measurements may have been insufficient in capturing the true experimental effects of reappraisal interventions to date. Finally, this study is limited to the examination of brief, experimental manipulation of reappraisal interventions. Some beliefs and associations we hold in regards to stress may be pervasive and difficult to change, thereby requiring more intensive interventions. These findings should be interpreted carefully and do not reflect on the efficacy of lengthier reappraisal interventions used in other settings.

Taken together, results from the meta-analysis and systematic review of reappraisal interventions suggest a promising avenue for the effective management of stress and optimization of stress responses with a few stipulations. First, it is important to consider theoretical models that provide the rationale for efficacy of these interventions, and incorporate measures across various domains of stress responsivity (e.g., reactivity and regulatory responses) in order to capture the mechanisms of change. Second, the measures employed should have enough sensitivity to change in order to distinguish meaningful differences, if any, that may be present across study conditions. Third, the use of study protocols, such as stressor type, should be meaningful and practical in engaging participants in order to reflect the applicability of stress intervention research.

Cognitive reappraisal techniques have been shown to be effective across domains of emotion regulation [15, 16]. The mechanisms of change proposed by reappraisal interventions in stress responsivity should thus emphasize the regulatory nature of this approach. At its roots, the reappraisal intervention of stress is not intended as an intervention aimed at reducing physiological response to stress, but rather as a regulatory tool to attenuate distress and perhaps, aid in the effective management of future stressors [13]. These important considerations should be incorporated into future study designs employing this intervention in order to better understand the mechanisms that contribute to its efficacy.

## Supporting information

S1 Table Study rigour. (DOCX)

S1 File Comprehensive meta-analysis software data. (CMA)

S2 File PRISMA 2009 checklist. (DOC)
