# Supplementary material for: The efficacy of stress reappraisal interventions on stress responsivity: A meta-analysis and systematic review of existing evidence
Source: PLoS One. 2019 Feb 27;14(2):e0212854. doi: 10.1371/journal.pone.0212854 (PMC6392321; doi:10.1371/journal.pone.0212854)
Supplement: S1 Table — (DOCX) [file pone.0212854.s001.docx]

S1 Table. Study rigour

| **Article** | **Sample Size** | **Randomized** | **Measures** | **Information about Intervention** | **Proper Statistical Test** | **Total Score** |
| --- | --- | --- | --- | --- | --- | --- |
| Akinola et al. [36] | 2 | 2 | 1 | 2 | 1 | **8** |
| Beltzer et al. [37] | 2 | 2 | 1 | 2 | 2 | **9** |
| Bowlin [38] | 2 | 2 | 1 | 1 | 2 | **8** |
| Brooks [39] | 2 | 2 | 2 | 0 | 1 | **7** |
| Cohen & Mor [40] | 2 | 2 | 0 | 1 | 1 | **6** |
| Crum et al. [24] | 2 | 2 | 2 | 1 | 2 | **9** |
| Denson et al. [41] | 2 | 2 | 2 | 2 | 2 | **10** |
| Erazo [42] | 2 | 2 | 2 | 1 | 2 | **9** |
| Germain & Kangas [43] | 2 | 0 | 2 | 1 | 2 | **7** |
| Gong et al. [44] | 2 | 2 | 0 | 1 | 2 | **7** |
| Gross [16] | 2 | 2 | 2 | 1 | 1 | **8** |
| Jamieson et al. [21] | 2 | 1 | 1 | 2 | 1 | **7** |
| Jamieson et al. [12] | 1 | 2 | 2 | 2 | 2 | **9** |
| Jamieson et al. [22] | 1 | 1 | 1 | 2 | 2 | **7** |
| Kneeland et al. [45] | 2 | 0 | 0 | 1 | 2 | **5** |
| Liu et al. [6] | 1 | 2 | 2 | 1 | 2 | **8** |
| Moore et al. [46] | 1 | 2 | 1 | 2 | 1 | **7** |
| Popham [47] | 2 | 2 | 2 | 2 | 2 | **10** |
| Sammy et al. [48] | 2 | 2 | 2 | 2 | 2 | **10** |
| Wang et al. [49] | 2 | 1 | 0 | 0 | 2 | **5** |
| Woud et al. [50] | 2 | 0 | 0 | 2 | 2 | **6** |
| Zhan et al. [51] | 2 | 0 | 2 | 0 | 2 | **6** |
